# Investigation of In-Vitro Adaptation toward Sodium Bituminosulfonate in *Staphylococcus aureus*

**DOI:** 10.3390/microorganisms8121962

**Published:** 2020-12-10

**Authors:** Marko Blisse, Evgeny A. Idelevich, Karsten Becker

**Affiliations:** 1 Institute of Medical Microbiology, University Hospital Münster, 48149 Münster, Germany; m_blis01@uni-muenster.de (M.B.); Evgeny.Idelevich@med.uni-greifswald.de (E.A.I.); 2 Friedrich Loeffler-Institute of Medical Microbiology, University Medicine Greifswald, 17475 Greifswald, Germany

**Keywords:** sodium bituminosulfonate, fusidic acid, antimicrobial resistance, susceptibility testing, sub-inhibitory concentrations, linear gradient, staphylococci, methicillin-resistant *Staphylococcus aureus*

## Abstract

The global increase in antimicrobial resistance has revived the interest in “old” substances with antimicrobial activity such as sodium bituminosulfonate. However, for those “old” compounds, scientific studies are still sparse and the ones available do not mostly meet the current standards. Since this compound is used for topical applications, investigation of a potential increase in minimal inhibitory concentrations (MICs) is of particular importance. For selection of phenotypes with decreased susceptibility, a collection of 30 genetically diverse methicillin-susceptible (MSSA) and methicillin-resistant *Staphylococcus aureus* (MRSA) strains were cultured on bi-layered linear gradient agar plates containing sub-inhibitory concentrations of the active agents. The stability of phenotypes with increased MICs was determined by serial passage on agent-free medium. Within 10 passages, only slight and, in most cases, reversible increases in MSSA and MRSA MIC levels toward sodium bituminosulfonate were obtained. Fusidic acid, used as a control, showed exponential expansions in MIC based on mutations in the *fusA* gene (elongation factor G or EF-G) with no reduction during the recovery phase. The only marginal and largely reversible changes of *S. aureus* MICs after exposition to sodium bituminosulfonate indicate a low potential for resistance development.

## 1. Introduction

The accelerated emergence and spread of antimicrobial resistances (AMR) generate a plethora of problems and challenges for the healthcare systems, especially due to limited treatment options [1,2]. In 2017, the World Health Organization (WHO) expressed the urgency for discovery and development of new antibiotics to fight, especially those pathogens displaying a variety of drug resistances and being of high clinical relevance, such as *Enterobacteriaceae* or *Staphylococcus aureus* [3]. The overuse of antibiotics in medicine and farming and lacking interest of many of the major pharmaceutical companies for the development of new antibiotic classes cause a fast decline in substances available to treat and prevent infections, especially due to multi-drug resistant microorganisms [2,4]. In particular, the current pipeline in antibiotic development is mainly limited to the derivative segment and lacks establishment of branches focused on innovative antibiotics with low probabilities for cross-resistance [2,5].

As a consequence of these circumstances, the interest in and use of “old” antibiotics is increasing [6,7]. The problem most “old” substances face is the lack of robust scientific data and studies, which meet current research methods and standards [7]. Providing such research represents a vital part in bringing those substances back into the clinical routine and highlight their use as an alternative treatment option in bacteria displaying multiple AMR [7]. One of these “old” substances is ammonium bituminosulfonate, which was already introduced in 1882 as “Ichthyol” [8]. Its pale analogue was made known in the 1930s as “Ichthyol, light” [9,10]. However, the mechanism of the antimicrobial activity of bituminosulfonate is still unclear. These compounds are water-soluble substances obtained from the sulfur-rich oil shale and represent the two fractions, which are the pale sulfonated shale oil (PSSO) and the dark sulfonated shale oil (DSSO), from the dry distillation process [11]. The few available publications on bituminosulfonate involve a variety of microbial species in outdated research experiments and give only very limited insights on the in vitro mechanism and activity [12,13,14,15]. A recent publication by Idelevich and Becker focused on the in vitro activity of sodium bituminosulfonate within the scope of current recommendations for antimicrobial susceptibility testing (AST), revealing activity against Gram-positive pathogens [16].

Bituminosulfonate has been described as a well-tolerated substance with pre-dominant use as a topical agent in the treatment of dermatological diseases such as eczematous dermatitis, wounds, and skin infections [11,17,18,19]. However, the widespread and unrestricted use of topical antimicrobial agents, such as mupirocin and fusidic acid, has been associated with the swift selection of resistant strains, as described for *S. aureus* [20,21,22,23].

Here, we investigated the exposure of a genetically diverse collection of methicillin-susceptible and methicillin-resistant *S. aureus* strains to sub-inhibitory concentrations of sodium bituminosulfonate in-vitro to analyze a possible emergence of phenotypes, displaying persistent increases in minimal inhibitory concentration (MIC) using linear compound gradients.

## 2. Materials and Methods

### 2.1. Bacterial Strains

The screening for the most resistant phenotype through sub-inhibitory sodium bituminosulfonate concentrations using bi-layered linear gradient agars was performed on a selection of strains previously described in various publications [24,25,26,27]. The selection of 30 genetically diverse *S. aureus* strains (Table S3) consists of 12 methicillin-susceptible *S. aureus* (MSSA; of these, there are two penicillin-susceptible *S. aureus* (PSSA)) and 18 methicillin-resistant *S. aureus* (MRSA). This selection included the reference strains ATCC 25923 (PSSA), ATCC 29213 (MSSA), and ATCC 43300 (MRSA). For fusidic acid, the reference strain and, additionally, one clinical strain selected from the groups of PSSA, MSSA, and MRSA were tested.

### 2.2. Preparation of Bi-Layered Linear Gradient Agar Plates

Bi-layered linear gradient agar plates were prepared in a two-step procedure. In the first step, 15 mL of cation-adjusted Mueller-Hinton agar (CA-MHA, BD Diagnostics, Heidelberg, Germany) containing adequate concentrations of the antimicrobial agent were poured into sterile standard petri dishes (94 mm in diameter and 16 mm in height) and the dishes were placed with an elevation of 5 mm on one side. The agar was allowed to completely harden by setting the plates for approximately 15 min in the tilted position. Afterward, a second layer of 15 mL of CA-MHA agar containing no active antimicrobial agent was poured and the plates are placed flat for another approximate 15 min to allow the second layer to harden fully as well. After the second layer was fully hardened, the plates were inversely incubated for approximately 6 h to allow the linear gradient to build up. The concentration of the linear gradients was adjusted accordingly to maintain a boundary zone for growth and no growth around the middle of the plate. The overall concentrations on the gradient agars reached from 0 g/L to 2 g/L for sodium bituminosulfonate (Ichthyol^®^ light, Ichthyol-Gesellschaft, Hamburg, Germany) and 0 mg/L to 4 mg/L for fusidic acid (Sigma-Aldrich, Saint Louis, MO, USA).

### 2.3. Exposure to Sub-Inhibitory Concentrations to Select the Least Susceptible Phenotypes

The screening for the least susceptible phenotypes was performed by inoculation of a gradient agar plate with a 1:10 dilution of a bacterial suspension having the turbidity of a 0.5±0.03 McFarland standard (approximately 10^7^ CFU/mL) using a cotton swab. The inoculated plates were incubated at 36 ± 1 °C for 18 ± 2 h. After incubation, plates were stored at room temperature until the next passage was initiated later on the same plate. Subsequent inoculation was done by taking bacterial material from the boundary zone of the gradient agar to prepare the bacterial suspensions with a turbidity of McFarland 0.5 ± 0.03. This procedure was repeated until 10 consecutive passages were performed. After 10 passages on gradient agars containing sub-inhibitory concentrations of the investigated antimicrobial agents, five additional passages on agent-free CA-MHA plates were performed and incubated under the same conditions as previously described.

### 2.4. Antimicrobial Susceptibility Testing

To identify changes in susceptibility against the tested antimicrobial agents, the ASTs were ascertained prior to the experiment and after each performed passage using the broth microdilution method, according to the recommendations of the Clinical and Laboratory Standards Institute (CLSI) and the International Organization for Standardization (ISO) [28,29,30]. Sodium bituminosulfonate was tested in double dilution concentration steps ranging from 0.008 g/L to 256 g/L and fusidic acid ranging from 0.008 mg/L to 256 mg/L. In brief, bacterial suspensions with the turbidity of a 0.5±0.03 McFarland standard were prepared from colonies of the boundary growth zone from gradient agar plates and diluted in cation-adjusted Mueller-Hinton broth (CA-MHB, BD Diagnostics, Heidelberg, Germany) to achieve a final inoculum in a test of approximately 5 × 10^5^ CFU/mL. Real bacterial concentration was confirmed by the colony counting of serial dilutions. The microtiter plates were incubated at 36 °C and read visually after 18 ± 2 h. In cases where trailing occurred, the sample was considered as negative if the growth was approximately ≤20% of the positive control.

### 2.5. Genetic Analysis of Fusidic Acid Resistance

For the genetic analysis, a selection of duplicates with different endpoint MICs after the five recovery passages were chosen from all tested strains. Total DNA was prepared using a QIAamp DNA Mini Kit (QIAamp DNA minikit; Qiagen, Hilden, Germany) following the manufacturer’s protocol.

The PCR primer and reaction conditions were applied as described by Chen et al. [31] and the generated amplicons were sent for sequencing (Eurofins Genomics Germany GmbH, Ebersberg, Germany). The results were analyzed with ClustalW alignment tool and graphically rendered using the Geneious software (Biomatters Ltd., Auckland, New Zealand).

## 3. Results

### 3.1. Baseline Assessment of MIC Values

The basal MIC values of sodium bituminosulfonate for the 30 *S. aureus* strains ranged from 0.06 to 0.25 g/L (median = 0.125 g/L) and exhibited a MIC_90_ of 0.25 g/L (Table 1 and Table S1). For fusidic acid, baseline MICs of the six tested *S. aureus* strains showed a range from 0.06 to 0.125 mg/L exhibiting MIC_90_ of 0.125 mg/L (Table 1 and Table S2).

### 3.2. Exposure to Sub-Inhibitory Concentrations to Select Least Susceptible Phenotypes

**Figure 1:**
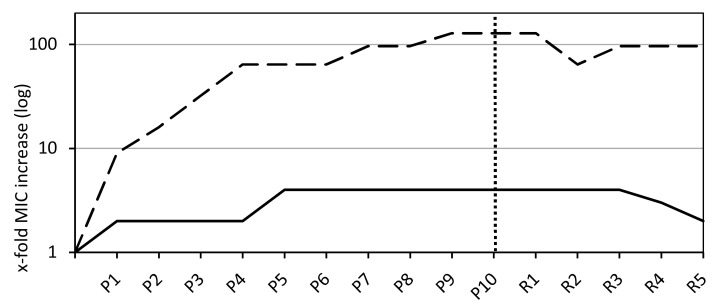
Minimal inhibitory concentration (MIC) values as a median of all tested *S. aureus* isolates for sodium bituminosulfonate (n = 30) and fusidic acid (n = 6, dashed line). The dotted vertical line indicates the endpoint after 10 passages on linear gradient plates containing the active agents (labeled with P) and recovery passages on plain agar plates (labeled with R).

Over the course of 10 passages on agar plates containing a linear gradient of sodium bituminosulfonate, slight and gradual increases of MICs were obtained within the first five passages followed by a stable continuity in MIC levels (Figure 1). The increases in MIC ranged from 0.25 to 2 g/L (median = 1) and the MIC_90_ has increased to 1 g/L. The MIC values increased within a range of 2-fold to 16-fold (median = 4-fold) (Table 1). For fusidic acid as a control substance, all tested strains (n = 6) displayed a rapid and exponential increase in MIC levels within the first four passages followed by a flat progression in MIC values (Figure 1). The endpoint MIC values after 10 passages on gradient plates ranged from 1 to 16 mg/L (median = 12) and yielded a MIC_90_ of 16 mg/L. The MIC values increases ranged from 16-fold to 256-fold (median = 128-fold) (Table 1). No significant differences between PSSA, MSSA, and MRSA could be observed, and all strains showed an exponential MIC increase within the early passages, passing into a mostly flat progression (Figure S1). The only identifiable but minor difference is within the extent of the adaption process. MICs of fusidic acid for the PSSA and MSSA strains rose to an elevation of 128-fold, whereas MRSA MICs rose 256-fold (Figure S1).

### 3.3. Analyzing the Stability of Phenotypes with Increased MICs in Recovery Passages

The majority of strains exposed to sodium bituminosulfonate (26/30) showed a decrease in MIC levels within the five recovery passages by serial passage on agent-free agar medium, showing the initiation of the reversible process after the 3rd recovery passage (Figure 1). The decreases in MIC from the 10th passage to the 5th recovery passage ranged from 1-fold to 4-fold (median = 2-fold) and endpoint MIC levels ranged from 1-fold to 8-fold (median = 2-fold) above the basal MICs (Table 1).

Within the five recovery passages, no decrease, except for ATCC 25923 (2-fold), in MIC was documented for fusidic acid, giving the endpoint MIC values a range of between 32-256-fold (median = 96-fold) above the basal levels (Figure 1, Table 1, and Table S2).

### 3.4. Genetic Mechanisms of the Fusidic Acid Resistance

Sequencing results from the selection of strains showed several polymorphisms alongside some silent mutations in the *fusA* gene. The majority of polymorphisms were single amino acid changes located at several positions within the amino acid sequence and in one case polymorphisms occurring in two positions. Identified polymorphisms were V90I, G323D, T385N, P406L, F437Y, H438D, G451V, L456F, H457Y, V471D, P478Q, K495E, and A655P as well as the dual polymorphism of V90I and M605I (Figure S2). Screening results for *fusB*, *fusC*, and *fusD* were negative.

## 4. Discussion

Sodium bituminosulfonate is a water-soluble substance used for topical treatments since its introduction in the late 19th century [19]. Latest research on susceptibility toward sodium bituminosulfonate meeting current AST standards [16] filled a gap from in-vitro data obtained in the 19th and 20th century and re-confirmed the activity of the substance against a variety of gram-positive bacteria while giving updated insights for current epidemiological circumstances [12,13,14,15]. These data from the AST support the revival process of this “old” substance [16], but possible changes in susceptibility during topical administration of sodium bituminosulfonate were still unknown, hence, being the aim of this study.

Previous experiments using sub-inhibitory concentrations on linear gradient plates and a multistep procedure established this method as a tool for analyzing the rise of AMR during in vitro experiments and complemented the single step approach to identify the mutation frequency [32,33,34]. Unsuitable antibiotics could be identified with this method through large increases in MIC values over the course of 10 passages and no significant decline during the subsequent recovery passages on plain agar plates [32,33,34,35]. The gained experimental data led to the advice for the reduction of those substances in therapeutic measures wherever possible or to the recommendation against using them as monotherapy to prevent further rise and spread of AMR. For instance, this was done for the 1962 introduced fusidic acid [36] after similar in vitro experiments indicated its tendency of AMR development during a single step event related to the inoculum density and the used concentration, making it a suitable control substance for our study [35,36,37,38].

*S. aureus* is one of the most frequent and significant pathogens of skin and soft tissue infections [39], often aggravated by methicillin-resistant strains. Thus, a genetically diverse collection of MRSA and MSSA were used for analyzing possible adaptation through decreased susceptibility toward sodium bituminosulfonate. Results from testing these strains to sub-inhibitory concentrations of sodium bituminosulfonate indicate a very low potential for resistance development. No exponential expansion in MIC values could be obtained during the 10 passages with linear gradient plates. Generated phenotypes exhibiting a lower susceptibility after 10 passages were not stable and 26 of 30 strains showed a reversion of the adaption process during the recovery passages on plain agar plates. A reversion toward baseline MIC levels ought to be missing, if a mechanism of AMR would have been induced, which was the case after application of fusidic acid as a control substance. All generated phenotypes lacking this reversion in MIC values during the recovery passages did not exhibit exponential MIC increase and MIC elevations that were only low to moderate. In contrast to sodium bituminosulfonate, fusidic acid as a comparator substance showed a quick and exponential expansion phase in MIC values within a short number of passages, indicating the development of AMR. Strains tested with fusidic acid displayed no reversion in MIC values on plain agar plates during the recovery passages and endpoint MICs were significantly above the clinical resistance breakpoint defined by the European Committee on Antimicrobial Susceptibility Testing (EUCAST) [40]. Since the dominant mechanism of AMR against fusidic acid is well described in the literature and mainly driven by mutations within the elongation factor G (EF-G), also known as the *fusA* gene, and related proteins interacting with EF-G, a genetic screening targeting these genes was used for validating previously described observations [31,41,42,43]. The found polymorphisms within the *fusA* gene verified the assumptions that the obtained exponential expansion in MIC values is a result of AMR of *S. aureus* toward fusidic acid. These findings underline the recommendations to avoid the administration of this antibiotic as monotherapy and for wide topical use and verify the methodology applied in this study [35,37,44].

Additionally, for the lack of evidence of a stable adaptation process resulting in decreased susceptibility toward sodium bituminosulfonate, it should be mentioned that the obtained MIC values are far below the concentrations in commercially available formulations. Currently sold formulations typically range from 6 g/L (Solutio Cordes^®^ 0.5%) to 240 g/L (Ichthotop^®^ 20%) of sodium bituminosulfonate. Since sodium bituminosulfonate is well tolerated [12,13,14], even undiluted concentrations up to 1200 g/L (liquid with a relative density of 1.2 g/mL) could be used for topical application and, thereby, leave more options to treat infections, even as bacterial adaptation processes might rise.

In conclusion, our in vitro experiments indicate a low potential for the resistance development toward sodium bituminosulfonate, making this “old” substance a continuing option for the topical treatment of *S. aureus* infections or for the eradication of cutaneous colonization by *S. aureus*.

## Figures and Tables

**Table 1:** Minimal inhibitory concentration (MIC) values and corresponding x-fold MIC increases for sodium bituminosulfonate and fusidic acid prior (baseline) and after 10 passages on agar plates containing the active agent as well as after five recovery passages on plain plates.

		MIC Values			x-Fold MIC Increase	
		Baseline	After 10 Passages	After 5 Recovery Passages	After 10 Passages	After 5 Recovery Passages
Sodium bituminosulfonate (n = 30)	MIC_90_	0.25 g/L	1 g/L	0.5 g/L	-	-
	Median	0.125 g/L	1 g/L	0.25 g/L	4	2
	Range	0.06–0.25 g/L	0.25–2 g/L	0.125–1 g/L	2–16	1–8
Fusidic acid (n = 6)	MIC_90_	0.125 mg/L	16 mg/L	16 mg/L	-	-
	Median	0.094 mg/L	12 mg/L	8 mg/L	128	96
	Range	0.06–0.125 mg/L	1–16 mg/L	2–16 mg/L	16–256	32–256

## Supplementary Materials

The following are available online at https://www.mdpi.com/2076-2607/8/12/1962/s1. Figure S1: Data from the penicillin-susceptible (A), methicillin-susceptible (B), and methicillin-resistant (C) reference strains exposed to 10 passages of sodium bituminosulfonate (solid line) and fusidic acid (dashed line) on linear gradient plates. The dotted vertical line indicates the endpoint after 10 passages on linear gradient plates containing the active agents (labeled with P) and five recovery passages on plain agar plates (labeled with R). Figure S2: Pairwise alignment of obtained polymorphisms within the *fusA* gene from the screening for a resistance mechanism. Table S1: Minimal inhibitory concentrations (MIC) values of the strains from the exposure to sub-inhibitory sodium bituminosulfonate concentrations over the course of 10 passages on the active agent and after five recovery passages on plain plates (labeled with R). All MIC values in g/L. Table S2: MIC values of the strains from the exposure to sub-inhibitory fusidic acid concentrations over the course of 10 passages on the active agent and after five recovery passages on plain plates (labeled with R). All MIC values in mg/L. Table S3: Clinical strain collection used in the study.
