# Supplementary material for: Investigation of In-Vitro Adaptation toward Sodium Bituminosulfonate in Staphylococcus aureus
Source: Microorganisms. 2020 Dec 10;8(12):1962. doi: 10.3390/microorganisms8121962 (PMC7763802; doi:10.3390/microorganisms8121962)
Supplement: Supplementary file 1 [file microorganisms-08-01962-s001.pdf]

Table S1. Minimum inhibitory concentration (MIC) values of the strains from the exposure to sub-inhibitory sodium bituminosulfonate concentrations over the course of ten passages on the active agent and after five recovery passages on plain plates (labeled with R). All MIC values in g/L.

Strain	MIC values determined after incubation of the strains on															
	Agar plates containing sub-inhibitory sodium bituminosulfonate concentrations											Plain agar plates				
	**Baseline**	**P1[a]**	**P2**	**P3**	**P4**	**P5**	**P6**	**P7**	**P8**	**P9**	**P10**	**R1[b]**	**R2**	**R3**	**R4**	**R5**
ATCC 29213	0.125	0.125	0.25	0.5	0.5	0.5	0.5	0.5	1	1	1	1	1	1	0.5	0.5
ATCC 25923	0.25	0.25	0.25	0.25	0.5	0.5	0.5	0.5	1	1	1	0.5	0.5	0.5	0.25	0.5
ATCC 43400	0.125	0.125	0.25	0.25	0.25	0.25	0.5	0.5	0.5	0.5	0.5	0.5	0.5	0.25	0.25	0.25
MOP4	0.25	1	1	1	1	2	1	2	2	1	2	1	1	1	1	0.5
MOP2	0.125	0.25	0.25	0.5	0.5	1	0.5	0.5	1	0.5	0.5	0.5	0.5	0.5	0.5	0.5
MOP12	0.125	0.25	0.5	0.5	1	1	1	1	1	0.5	1	1	0.5	0.25	0.25	0.5
MOP14	0.06	0.125	0.25	0.25	0.5	0.5	0.5	0.5	0.5	0.5	1	0.5	0.5	0.5	0.5	0.5
MOP16	0.25	0.5	0.5	1	0.5	1	1	1	1	1	1	1	1	1	1	1
MOP18	0.06	0.125	0.25	0.25	0.25	0.25	0.25	0.25	0.25	0.5	0.25	0.25	0.25	0.25	0.25	0.25
MOP20	0.125	0.25	0.5	1	0.5	1	0.5	1	1	1	1	1	1	1	0.5	0.5
MOP22	0.06	0.125	0.25	0.5	0.5	0.5	0.25	0.5	0.5	0.5	0.5	0.5	0.25	0.25	0.5	0.25
MOP24	0.06	0.125	0.25	0.5	0.25	0.5	0.5	0.5	0.5	0.5	0.5	0.5	0.5	0.5	0.5	0.5
MOP26	0.125	0.125	0.125	0.25	0.25	0.25	0.25	0.25	0.25	0.25	0.5	0.5	0.25	0.25	0.25	0.25
11–03	0.25	0.25	0.25	0.5	0.5	0.25	0.5	0.5	0.5	1	1	1	0.5	0.5	0.5	0.5
11–15	0.25	0.25	0.25	0.5	0.25	0.25	0.25	0.5	0.5	0.5	1	0.5	0.5	0.25	0.25	0.25
11–12	0.06	0.06	0.06	0.125	0.125	0.06	0.125	0.125	0.125	0.25	0.5	0.5	0.5	0.25	0.25	0.25
10–50	0.25	0.25	0.25	0.5	0.25	0.25	0.5	0.5	0.5	0.5	0.5	0.5	0.5	0.5	0.25	0.25
09–32	0.25	0.25	0.25	0.25	0.25	0.5	0.25	0.5	0.5	0.5	0.5	0.25	0.25	0.25	0.25	0.25
11–04	0.125	0.25	0.25	0.25	0.25	0.25	0.25	0.25	0.25	0.25	0.5	0.5	0.25	0.25	0.25	0.25
17–48	0.25	0.25	0.25	0.25	0.5	0.5	0.5	0.5	1	1	1	1	1	1	1	0.5
10–17	0.25	0.25	0.25	0.5	0.25	0.25	0.25	0.5	0.5	0.5	1	0.5	0.5	0.5	0.25	0.25
09–26	0.125	0.25	0.25	0.25	0.25	0.25	0.25	0.25	0.5	0.5	0.5	0.5	0.5	0.5	0.5	0.25
13–02	0.125	0.25	0.25	0.25	0.25	0.5	0.25	0.5	0.5	1	0.5	0.25	0.5	0.25	0.25	0.25
11–31	0.125	0.25	0.25	0.25	0.25	0.5	0.5	0.5	1	1	1	0.5	0.5	0.25	0.25	0.25
30–38	0.25	0.25	0.25	0.25	0.25	0.25	0.5	0.5	1	1	1	1	0.5	1	0.5	0.25
20–35	0.125	0.125	0.25	0.25	0.5	0.25	0.5	0.25	0.5	0.5	0.25	0.25	0.25	0.25	0.125	0.125
LM380270	0.125	0.5	0.5	1	0.5	1	1	1	1	1	1	1	0.5	0.5	0.5	0.25
LM379438	0.125	0.25	0.5	0.25	0.25	0.5	0.5	0.5	1	1	1	0.5	0.5	0.5	0.25	0.25
LM297503	0.125	0.25	0.5	0.5	0.5	0.5	0.5	0.5	1	1	1	1	0.5	0.5	0.5	0.25
023–7049	0.125	0.25	0.5	1	1	1	1	1	1	1	1	0.5	0.5	0.5	0.5	0.25

[a]Passages on plates containing a linear gradient of the microbial agent. [b]Passages on plain agar plates.

Table S2. MIC values of the strains from the exposure to sub-inhibitory fusidic acid concentrations over the course of ten passages on the active agent and after five recovery passages on plain plates (labeled with R). All MIC values in mg/L.

Strain	MIC values determined after incubation of the strains on															
	Agar plates containing sub-inhibitory fusidic acid concentrations											Plain agar plates				
	**Baseline**	**P1**	**P2**	**P3**	**P4**	**P5**	**P6**	**P7**	**P8**	**P9**	**P10**	**R1**	**R2**	**R3**	**R4**	**R5**
ATCC 29213	0.125	2	8	16	8	8	8	16	16	16	16	16	16	16	16	16
ATCC 25923	0.125	2	2	2	8	8	8	8	8	16	16	8	8	16	8	8
ATCC 43300	0.06	8	8	16	16	8	16	16	16	16	16	16	16	16	16	16
MOP4	0.06	0.125	0.25	2	4	4	8	8	8	8	8	8	4	4	8	8
MOP2	0.06	0.125	0.25	0.5	2	1	1	2	1	1	1	2	2	2	2	2
11–03	0.125	0.25	2	4	4	4	4	2	4	8	8	16	8	8	8	8

[a]Passages on plates containing a linear gradient of the microbial agent. [b]Passages on plain agar plates.

Table S3. Clinical strain collection used in the study.

Strain	*spa*-type	Methicillin resistance phenotype (*mec* gene type)	Source
MOP4	t150	PSSA-MSSA (n.d.)	[33]
MOP2	(11–17–12–17)	PRSA-MSSA (n.d.)	[33]
MOP12	t002	PRSA-MSSA (n.d.)	[33]
MOP14	t1909	PRSA-MSSA (n.d.)	[33]
MOP16	t084	PRSA-MSSA (n.d.)	[33]
MOP18	t021	PRSA-MSSA (n.d.)	[33]
MOP20	t548	PRSA-MSSA (n.d.)	[33]
MOP22	t949	PRSA-MSSA (n.d.)	[33]
MOP24	t298	PRSA-MSSA (n.d.)	[33]
MOP26	t015	PRSA-MSSA (n.d.)	[33]
11–03	t003	HA-MRSA (*mecA*)	[34]
11–15	t032	HA-MRSA (*mecA*)	[34]
11–12	t008	HA-MRSA (*mecA*)	[34]
10–50	t002	HA-MRSA (*mecA*)	[34]
09–32	t045	HA-MRSA (*mecA*)	[34]
11–04	t004	HA-MRSA (*mecA*)	[34]
17–48	t014	HA-MRSA (*mecA*)	[34]
10–17	t041	HA-MRSA (*mecA*)	[34]
09–26	t022	HA-MRSA (*mecA*)	[34]
13–02	t038	HA-MRSA (*mecA*)	[34]
11–31	t044	PVL-positive CA-MRSA (*mecA*)	[34]
30–38	t008	PVL-positive CA-MRSA (*mecA*)	[34]
20–35	t693	PVL-positive CA-MRSA (*mecA*)	[34]
LM380270	t011	LA-MRSA (*mecA*)	[35]
LM379438	t034	LA-MRSA (*mecA*)	[35]
LM297503	t108	LA-MRSA (*mecA*)	[35]
023–7049	t843	LA-MRSA (*mecC*)	[36]

MSSA, methicillin-susceptible *S. aureus*; PSSA, penicillin-susceptible *S. aureus*; PRSA, penicillin-resistant *S. aureus*; MRSA, methicillin-resistant *S. aureus*; HA-MRSA, healthcare-associated *S. aureus*; CA-MRSA, community acquired *S. aureus*; PVL-pos, Panton Valentine leucocidin positive; LA-MRSA, livestock associated *S. aureus*; n.d., not detected.

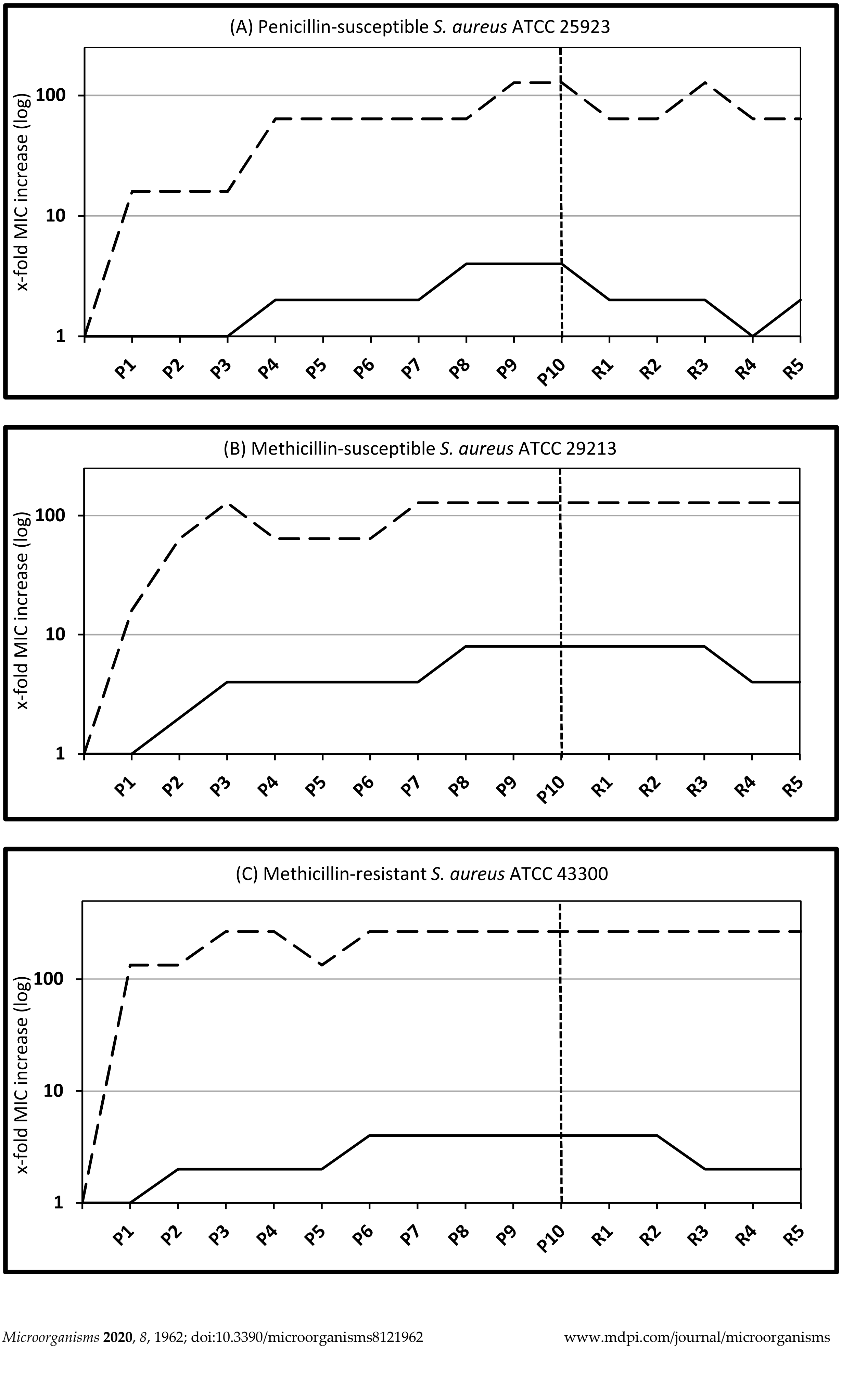
(A) Penicillin-susceptible *S. aureus* ATCC 25923
x-fold MIC increase (log)
100
10
1
P1 P2 P3 P4 P5 P6 P7 P8 P9 P10 R1 R2 R3 R4 R5
(B) Methicillin-susceptible *S. aureus* ATCC 29213
x-fold MIC increase (log)
100
10
1
P1 P2 P3 P4 P5 P6 P7 P8 P9 P10 R1 R2 R3 R4 R5
(C) Methicillin-resistant *S. aureus* ATCC 43300
x-fold MIC increase (log)
100
10
1
P1 P2 P3 P4 P5 P6 P7 P8 P9 P10 R1 R2 R3 R4 R5

Figure S1. Data from the penicillin-susceptible (**A**), methicillin-susceptible (**B**) and methicillin-resistant (**C**) reference strains exposed to ten passages of sodium bituminosulfonate (solid line) and fusidic acid (dashed line) on linear gradient plates. The dotted vertical line indicates the endpoint after ten passages on linear gradient plates containing the active agents (labeled with P) and five recovery passages on plain agar plates (labeled with R).

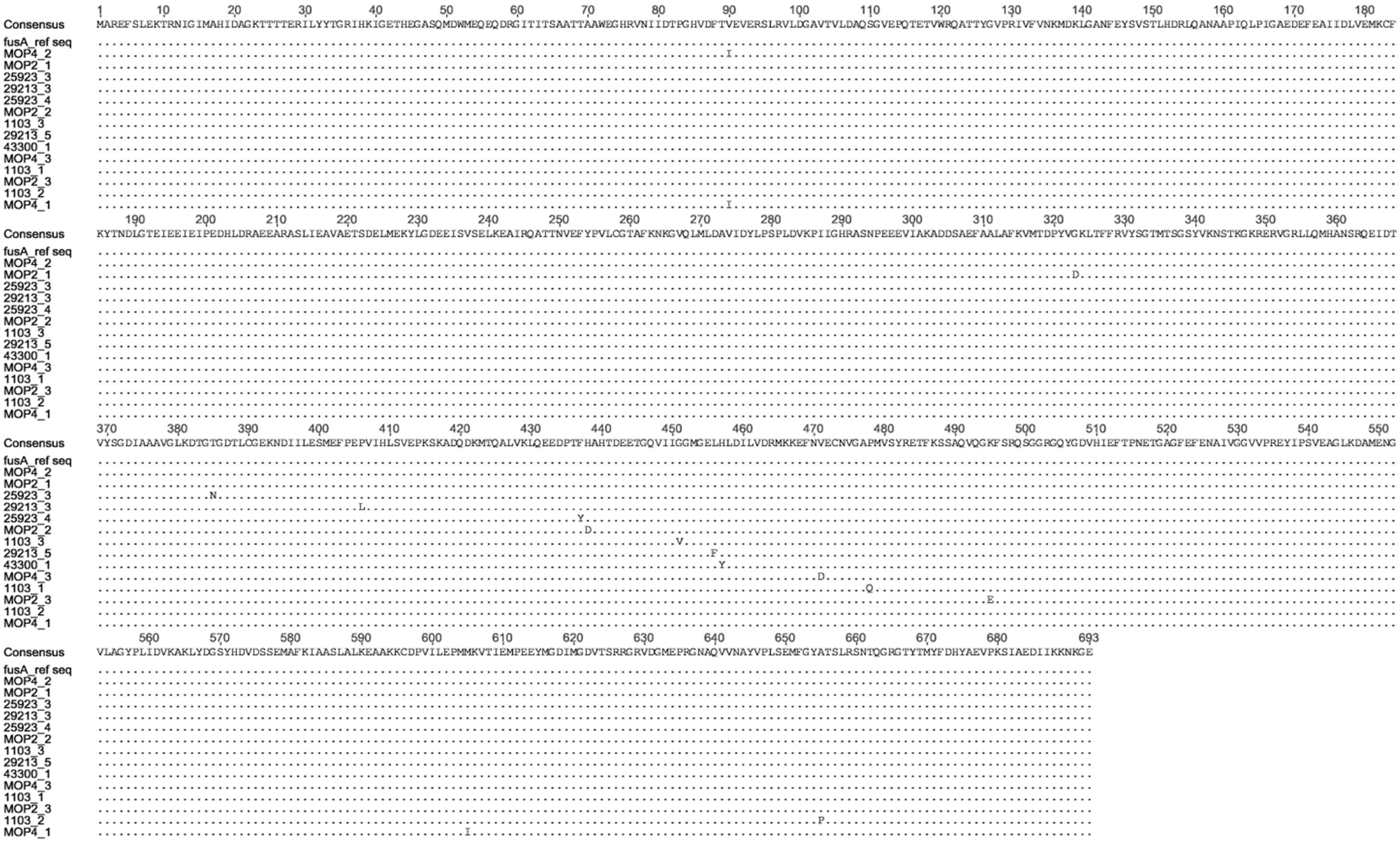

Figure S2. Pairwise alignment of obtained polymorphisms within the *fusA* gene from the screening for resistance mechanism.

© 2020 by the author. Licensee MDPI, Basel, Switzerland. This article is an open access article distributed under the terms and conditions of the Creative Commons Attribution (CC BY) license (http://creativecommons.org/licenses/by/4.0/).
